# Association between Overweight/Obesity and the Safety and Efficacy of COVID-19 Vaccination: A Systematic Review

**DOI:** 10.3390/vaccines11050996

**Published:** 2023-05-17

**Authors:** Cangcang Fu, Nan Lin, Jihua Zhu, Qing Ye

**Affiliations:** Children’s Hospital, Zhejiang University School of Medicine, National Clinical Research Center for Child Health, Hangzhou 310052, China

**Keywords:** COVID-19, vaccine, obesity, overweight, review

## Abstract

Objective: The objective of this study was to appraise the interrelation between overweight/obesity and the safety and efficacy of COVID-19 vaccination by synthesizing the currently available evidence. Methods: A systematic review of published studies on the safety and efficacy of the COVID-19 vaccine in people who were overweight or obese was conducted. Databases including Embase, Medline Epub (Ovid), PsychInfo (Ovid), Web of Science, PubMed, CINAHL, and Google Scholar were searched to identify relevant studies. The databases of the Centers for Disease Control (CDC) and World Health Organization (WHO) were also searched for relevant unpublished and gray literature. Results: Fifteen studies were included in the review. All the included studies used observational study designs; there were ten cohort studies and five cross-sectional studies. The sample size of these studies ranged from 21 to 9,171,524. Thirteen studies reported using BNT162b2 (Pfizer-BioNTech, USA), four reported using ChAdOx-nCov19 (AstraZeneca, U.K), two were reported using CoronaVac (Sinovac, China), and two were reported using mRNA1273 (Moderna, USA). The efficacy and safety of COVID-19 vaccines have been extensively studied in individuals with overweight/obesity. Most studies have shown that the humoral response decreases with increasing BMI. The available evidence does not conclusively indicate that these vaccines are generally safe in this population. Conclusion: While the efficacy of the COVID-19 vaccine may be less than ideal in people who are overweight or obese, it does not mean that obese people should not be vaccinated, as the vaccine can still provide some protection. There is a lack of evidence for conclusions to be drawn about the safety of the vaccine in the population. This study calls on health professionals, policymakers, caregivers, and all other stakeholders to focus on monitoring the possible adverse effects of injections in overweight/obese people.

## 1. Introduction

Since the beginning of the coronavirus disease 2019 (COVID-19) pandemic, SARS-CoV-2 has rapidly spread and caused over 656 million confirmed cases and over 6.6 million deaths globally, as reported by the WHO on 1 January 2023 [1]. COVID-19 has caused widespread damage and trauma to people, but the epidemic is still not under control. COVID-19 vaccines, including inactivated vaccines, live-attenuated vaccines, viral vector vaccines, and mRNA vaccines, are effective in preventing and controlling COVID-19 infections by stimulating the human immune system to produce specific antibodies to generate protection for the immunized individual [2]. Although the emergence of SARS-CoV-2 variants, which appear to be more transmissible and may escape from convalescent immune responses, has slowed the progress of controlling the pandemic [3], vaccinations and booster doses can still curb the spread of the disease and potentially help to achieve herd immunity [4].

Promoting vaccination is crucial for ending the spread of COVID-19. There were twenty-four COVID-19 vaccines approved by the WHO Emergency Use Listing Procedure worldwide and 176 candidates in clinical development processes as of 1 January 2023 [5]. Over 130 billion vaccine doses had been administered globally as of 21 December 2022 [6]. Despite the availability of vaccination against COVID-19 for all populations [4], there is still limited evidence of its safety and efficiency among people who are overweight and obese [7,8]. Obesity is one of the most serious health concerns because it impairs the immune system and leads to a variety of diseases, such as diabetes, cardiovascular disease, and nonalcoholic liver disease [9]. Claiming to critically evaluate the scientific data from the published peer-reviewed literature and briefing documents available at the time, the Obesity Society claimed that vaccines from Pfizer-BioNTech, Moderna, and Johnson & Johnson are highly effective, independently from weight status [10]. However, several studies on immunogenicity have mentioned that COVID-19 vaccinations for people with overweight or obesity status might be less effective due to chronic inflammation and metabolic dysregulation [11,12,13,14], thus resulting in a greater risk of infection. Some studies suggest that obese people may need a higher dose of the vaccine than nonobese people to generate an adequate immune response [11,12]. Current studies have shown that the safety of COVID-19 vaccines in obese people is comparable to that in nonobese people, with no significant safety concerns being found [13]. However, there is no concrete evidence to establish this, as the sample sizes are limited and no long-term follow-up has been conducted. There is currently no consensus on the effectiveness of COVID-19 vaccines in obese people and whether infections occur. Research is needed to study the safety and efficiency of the population with overweight and obesity. Evidence on this issue is scarce. Therefore, we conducted a systematic review of the newly published, peer-reviewed literature and related documentation to assess the relationship between the safety and effectiveness of COVID-19 vaccination in obese people.

### Research Hypotheses and Objectives

The research hypothesis was as follows: “The COVID-19 vaccine may be less effective and safer in obese people”.

The objectives were as follows:

1. To explore the effectiveness of the COVID-19 vaccine in obese people.
2. To explore the safety of the COVID-19 vaccine in obese people.

## 2. Methods

### 2.1. Registration

This systematic review followed the Preferred Reporting Items for Systematic Reviews and Meta-Analysis (PRISMA) guidelines (available in the Supplementary Materials) [15]. The systematic review protocol was registered at PROSPERO (registration number: CRD42023426734).

### 2.2. Literature Search

A systematic literature search was conducted in Embase, Medline Epub (Ovid), PsychInfo (Ovid), Web of Science, PubMed, CINAHL, and Google Scholar to identify relevant studies.

Databases were searched from inception to January 2023. The following keywords and their synonyms were included in the search: “COVID-19 vaccine”, “SARS-CoV-2 vaccine”, “efficacy”, “safety,” “overweight”, “obese”, and “body mass index”. The search strategy was adapted for each database. Relevant reviews and the included articles were hand-searched for additional eligible studies. In addition, the databases of the Centers for Disease Control (CDC) and World Health Organization (WHO) were also searched for relevant unpublished and gray literature. The Supplementary Material (File S1) describes the search strategies in their entirety.

### 2.3. Inclusion and Exclusion Criteria

The included studies met the following eligibility criteria: (1) participants were previously vaccinated for COVID-19; (2) they originated from randomized trials and observational studies (including cohort studies, case–control studies, cross-sectional study designs, and case reports); (3) they provided data regarding the association between overweight/obese and the safety (e.g., the incidence of adverse events) and efficacy (e.g., cases of (post-vaccination) SARS-CoV-2 infection) of the COVID-19 vaccine; (4) they were published in peer-reviewed journals; and (5) they were available in English. Studies were excluded for the following reasons: (1) studies on animals; (2) in vitro/ex vivo; and (3) qualitative studies, reviews, theses, conference papers, book chapters, news reports, and letters.

### 2.4. Selection Process

All references were exported and managed with Endnote X9. Title/abstract screening was independently performed by two reviewers (N.L. and C.F.) based on the inclusion criteria. Relevant articles were retrieved for full-text reading and further review by two reviewers (N.L. and C.F.). The two authors discussed disagreements until they agreed. The remaining disagreements were discussed with a third author (J.Z.) until a consensus was reached.

### 2.5. Data Extraction

Data from individual studies were extracted and organized with an extraction form by one reviewer (N.L.) and then verified by another (C.F.). The extracted information included (1) the basic characteristics of the included studies (first authors, year of publication, study design, location, and period), (2) the characteristics of participants (age, sex, sample size, indicators of overweight/obesity), (3) vaccines (vaccine name, platform, number of doses), (4) covariate adjustment, and (5) outcomes regarding the association between overweight/obesity and the safety (e.g., the incidence of overall, local, and systemic adverse events) and efficacy (e.g., number of (postvaccination) SARS-CoV-2 infections, hospitalization for COVID-19, and admission to the ICU for COVID-19) of the COVID-19 vaccine. Effect estimates were extracted from the fully adjusted models (if available).

### 2.6. Assessment of Risk of Bias

Two reviewers independently assessed the risk of bias in the included studies by using the Joanna Briggs Institute (JBI) critical appraisal checklist [16]. The checklist allows for methodological and bias assessment in quantitative and qualitative analyses with varying study designs, including cohort studies and cross-sectional studies [17]. The response options of the checklist included “Yes” (the criteria are identifiable through the report description), “Unclear” (the criteria are not identified in the report), and “No” (the criteria are not identifiable). Based on the number (%) of “Yes” responses, the risk of bias was further rated as “high” (≤49%), “moderate” (50%−69%), and “low” (≥70%) [16]. All of the disagreements during the evaluation were later discussed, and a consensus was reached. There were no exclusions made based on a minimum threshold. The interrater reliability (IRR) test was performed with the Kappa calculator in the bias risk test. The percentage of user consent was calculated by dividing the number of studies in which both authors gave the same risk score for bias by the total number of studies. Cohen’s Kappa test was used to consider the same risk-of-bias scores, with Kappa values ranging from 0.457 to 1.

## 3. Results

### 3.1. Study Selection

**Figure 1:**
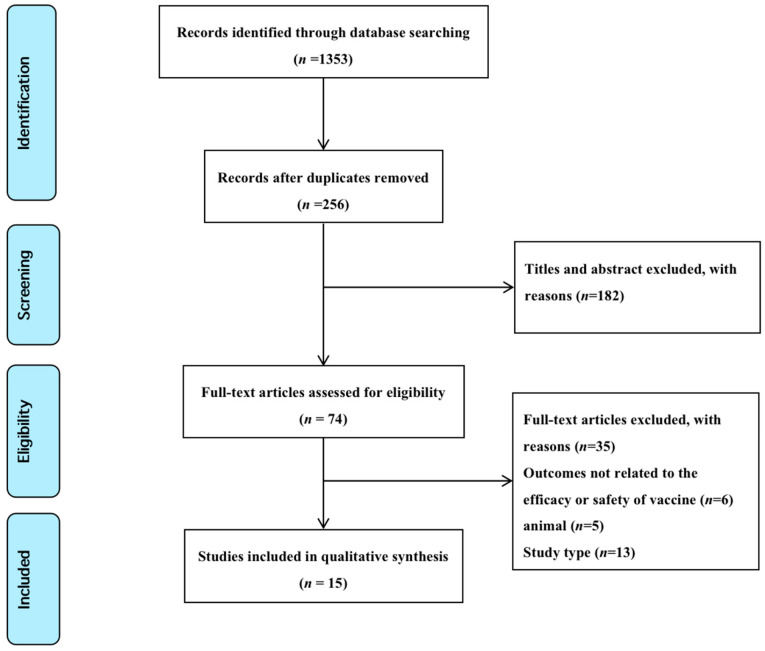
PRISMA flow diagram of the selection of the studies, which are listed in Table 1, and the characteristics of the included studies (*n* = 15).

One thousand three hundred and fifty-three studies were imported into EndNote software. A total of 1097 studies were removed based on the exclusion criteria during title/abstract screening. The remaining 74 studies were screened for full-text review. Of these, 59 were excluded because they did not meet the eligibility criteria. Therefore, 15 studies were included in the review [11,13,14,18,19,20,21,22,23,24,25,26,27,28,29] (Figure 1).

### 3.2. Study Characteristics

The characteristics of the included studies are described in Table 1. All included studies used observational study designs; there were ten cohort studies and five cross-sectional studies. The sample size of these studies ranged from 21 to 9,171,524. Thirteen studies reported using BNT162b2, four reported using ChAdOx-nCov19, two reported using CoronaVac, two reported using mRNA1273, and one study compared the safety between the Sinovac vaccine and the Pfizer vaccine.

### 3.3. Quality Appraisal

The risk-of-bias assessments for cohort studies, cross-sectional studies, and case series are summarized in Table S1 and Table S2 in the Supplementary Materials. Of the 15 included studies, ten had a ‘low’ risk of bias, four had a ‘moderate’ risk of bias, and one had a ‘high’ risk of bias.

### 3.4. Synthesis of Results

Six studies [13,19,26,27,28,29] investigated the association between overweight/obesity and the safety of COVID-19 vaccination. Study 3 [19] was a cohort study aimed at evaluating the safety of COVID-19 vaccine-induced humoral and cellular immune responses in Chinese individuals with obesity/overweight. The findings showed that inactivated COVID-19 vaccines were safe, and no serious vaccine-related adverse events occurred. Study 11 [26] was a national cohort study among 9,171,524 participants in England. Their results suggested that there were significant linear associations between BMI and COVID-19 hospitalization and death after the first dose and J-shaped associations after the second dose. However, Study 12 [13] was a cohort study that explored variables associated with the serological response following an mRNA COVID-19 vaccine in 86 healthcare workers and found that central obesity, such as that in people with a higher waist circumference, waist-to-hip ratio, BMI, or body fat, was not associated with more adverse events. Study 13 [27] was a cross-sectional study that investigated 2136 adults and found that nonoverweight status was associated with a higher risk of side effects, such as fever, vomiting, diarrhea, and chills, than overweight status. Study 14 [28] was a cohort study on the type and frequency of adverse reactions in healthy individuals and those with allergic disease aged 5–11 years over the first seven days following the first and second BNT162b2 vaccinations. The participants were recruited from a hospital and four municipalities in the Ishikawa district of Japan. The results of the logistic regression showed no statistically significant associations between BMI and experiencing adverse systemic reactions after seven days of vaccination, with odds ratios and 95% confidence intervals of 0.80 (0.46–1.40) (thin vs. normal) and 0.69 (0.42–1.13) (overweight vs. normal), respectively. Study 15 [29] was a cross-sectional study aimed at evaluating a comparison between the Sinovac vaccine and the Pfizer vaccine for children and teenagers under 18 years old and other factors that influenced it. The participants were a convenience sample of 400 children and teenagers who received the total doses of the Sinovac and Pfizer vaccines in Indonesia. Their findings suggested that vaccine recipients with a BMI of less than 25 had a higher risk of having side effects, including fever, pain in the injection area, lost smell and taste after the first vaccination, sleepiness and fever after the second vaccination, and menstrual problems after 1–3 months and 4–6 months postvaccination (*p* < 0.05).

### 3.5. Overweight/Obesity and COVID-19 Vaccine Efficacy

Among the 12 selected studies [11,13,14,18,19,20,21,22,23,24,25,26], eight cohort studies [11,13,14,18,19,23,24,25] provided absolute values of antibody titers in an obese group and nonobese group, and three studies [11,13,25] offered baseline antibody titers. When compared with the nonobese groups, the antibody titers of the obese groups were lower [11,13,14,18,19,20,22,23,24,26]. However, two studies [21,25] suggested that obesity was not associated with antibody responses after two doses of ChAdOx1 nCoV-19 vaccination. We carefully reviewed the full text of this article. It was found that the population was previously infected with COVID-19. There have also been studies examining obesity and vaccine effectiveness in children and adolescents. One study [14] from Thailand examined the efficacy of the COVID-19 vaccine among obese adolescents. In this cohort study, Tubjaroen [14] aimed to evaluate immunogenicity among children with liver transplants and obesity following two doses of the BNT162b2 vaccine. They observed that the SARS-CoV-2 antibody levels at approximately four weeks following the second dose of the BNT162b2 vaccine in obese adolescents were lower than those in healthy controls with normal weight (2999.4 ± 1725.9 vs. 4960.5 ± 644.1 IU/mL, *p* < 0.001). They had a slightly high inhibition of sVNT for the Delta variant up to 1 month after vaccination (96.6 ±.6% vs. 98.5 ± 1.6%, *p* = 0.06).

## 4. Discussion

The results of this study partially support the hypothesis of the study. The relationship between obesity and COVID-19 vaccination has been a subject of considerable attention and debate due to the potential influence of obesity on both severe COVID-19 disease and vaccine efficacy against infectious diseases [27]. Therefore, it is crucial to assess the potential impact of obesity on COVID-19 vaccinations. A longitudinal study was conducted to assess the impact of central obesity on the efficacy of Pfizer/BioNTech vaccination in a cohort of 86 healthcare workers in Italy [13]. The study revealed that lower antibody titers were associated with central obesity, independently from BMI. In another investigation, Pellini and colleagues [25] examined the antibody titers of individuals with a healthy weight, overweight, or obesity between the first and second doses of the Pfizer/BioNTech vaccine. They observed that a humoral immune response was activated by a single vaccination in individuals with healthy weight, while some overweight or obese subjects (age > 47 and BMI > 25 kg/m^2^) did not experience a change in their IgG antibody levels. Nine of the twelve studies that investigated the humoral response reported a reduced response with increasing BMI. Overall, BMI was associated with a higher initial increase in IgG antibodies after immunization, and a higher BMI was associated with a greater decline in antibody titers. This may be because obesity can lead to inflammation and immune dysfunction, which can affect the body’s immune response after vaccination, thus reducing its protective effect. However, most of the studies did not follow up for more than 12 months, so more studies are needed to better understand the long-term efficacy of COVID-19 immunization in obese populations.

Despite the possible effects of obesity on vaccine efficacy, it is noteworthy that vaccination is still recommended for overweight or obese individuals. The benefits of vaccination, such as reductions in severe COVID-19 disease and hospitalization, outweigh the potential risks associated with a weaker immune response in this population [26,30]. Furthermore, addressing the root causes of obesity may enhance individuals’ overall health and potentially improve their immune responses to the vaccine [31]. Implementing strategies for encouraging healthy eating habits, physical activity, and weight reduction could increase vaccine efficacy and lower the risk of severe COVID-19 infections in this population [22].

Vaccination is considered the most effective tool for controlling the spread of COVID-19 and reducing severe outcomes. However, it remains unclear how overweight/obesity, a common comorbidity, affects the safety of COVID-19 vaccination [32]. Several studies have investigated the safety of COVID-19 vaccination in individuals with overweight/obesity [13,19,26,27,28,29]. Three studies suggested that COVID-19 vaccines have been shown to be safe in people with overweight/obesity, and there was no direct link between BMI and vaccine side effects [13,19,28]. However, a study focused on adolescents suggested that the association between BMI and COVID-19 vaccine side effects may vary with dose and time [29]. Interestingly, two studies showed that nonobese individuals were more likely to experience side effects than obese individuals [27,29]. A national study reported more adverse effects at the high and low extremes of BMI [26]. The immune system’s response to the vaccine is not significantly affected by obesity in most cases, so there might be no increased risk of side effects for individuals who are overweight or obese. Underweight individuals may have weaker immune systems due to malnutrition or underlying health conditions, making them more susceptible to vaccine side effects [33]. The relationship between body weight and vaccine side effects may be influenced by other factors, such as age, sex, or underlying health conditions. Therefore, the available evidence is not yet conclusive, and further research is needed to investigate the optimal vaccine regimen for this population.

Our study has some limitations. First, there are only a limited number of studies on COVID-19 vaccines and obesity, most of which have small and limited sample sizes and large heterogeneity, making it difficult to reach research conclusions. Consequently, there is usually a different set of comorbidities associated with adults with obesity when compared with children with obesity. Comparing both groups without much consideration for these confounders or stratifying by the age group would in itself contribute to some bias.

## 5. Conclusions

In conclusion, the association between obesity and COVID-19 vaccination warrants further investigation, as it could impact vaccine efficacy and the overall health outcomes for this population. This study found that the humoral response decreased with increasing BMI, but this result may slightly vary by gender, vaccine dose, and comorbidity. Moreover, the safety of novel coronavirus vaccines cannot be concluded because the results vary too widely from study to study. Interestingly, this study found that novel coronavirus vaccines may cause more side effects in nonobese people, which also provides some new ideas for the design and focus of follow-up studies. Implementing strategies to address the underlying causes of obesity could also enhance vaccine efficacy and overall health outcomes.

## Figures and Tables

**Table 1:** Characteristics of the included studies.

No.	1st Author (Year)	Study Objectives	Country	Study Design	Population	Follow-up Period	Vaccine Received	Outcomes	Conclusion
1	Malavazos AE,2022 [11]	To evaluate how people with AO respond to mRNA vaccines against SARS-CoV-2	Italy	cohort study	healthcare workers; AO = 492, NoAO = 568; Age: 41.42 ± 12.95	(1) Baseline (2) 21 days after dose 1 (3) 1 month after dose 2 (4) 3 months after dose 2	BNT162b2	Vaccine Efficacy: Between the first and third month after vaccine dose 2, the drop in IgG-TrimericS levels was more remarkable in individuals with AO compared with those without AO	The waning antibody levels in individuals with AO
2	Gaborit B,2023 [18]	To investigate early humoral response to COVID-19 vaccination in patients with obesity	France	cohort study	patients with obesity (*n* = 357) healthy subjects (*n* = 573); Age: 47.2 ± 14.7	1, 6, 12, and 24 months after the first two doses of COVID-19 vaccines	(1) BNT162b2 (2) mRNA-1273 (3) ChAdOxnCov-2019	Vaccine Efficacy: Patients with obesity were less likely to have positive antibodies after the first and second doses compared to the controls	The humoral response to the COVID-19 vaccine was lower in patients with obesity one month after the second dose
3	Zhu Q,2022 [19]	To evaluate the safety profile and provide new insights into inactivated COVID-19-vaccine-induced humoral and cellular immune responses	China	cohort study	obesity/overweight (*n* = 132) normal BMI (*n* = 82); Age: 18 to 75 years	21−105 days after full-course COVID-19 vaccination	BBIBP-CorV/CoronaVac	Vaccine Safety: No serious vaccine-related adverse effects occurred. Vaccine Efficacy: Individuals with obesity/overweight had lower NAbs Anti-RBD-IgG	Inactivated COVID-19 vaccines were safe and well tolerated but induced poor humoral and cellular immune responses in individuals with obesity/overweight
4	Faizo AA,2023 [20]	To investigate the effectiveness of COVID-19 vaccines among individuals with obesity	Jeddah, Saudi Arabia	cross-sectional study	obese (*n* = 73) normal BMI (*n* = 46); Age: 18 to >60 years	(1) <90 days (2) 90–180 days (3) >180 days	(1) BNT162b2 (2) ChAdOx nCov-2019 (3) mRNA-1273	Vaccine Efficacy: The observed reduction in COVID-19-vaccine-induced neutralizing humoral immunity among obese individuals occurred independently of gender, recovery from past infection, and time since the last vaccination.	The effectiveness of COVID-19 vaccines is potentially reduced among obese individuals
5	Lee SW, 2021 [21]	To assess the relationships of antibody level with BMI	Korea	cross-sectional study	healthcare workers; Obese (*n* = 88); Age: 40.6 ± 10.9	(1) 7 days after each injection (2) 4 weeks after the second injection	ChAdOx1 nCoV-19	Vaccine Efficacy: Anti-SARS-CoV-2 S protein RBD concentration showed no significant association of antibody concentration with BMI	Obesity was not associated with antibody responses after two doses of ChAdOx1 nCoV-19 vaccination
6	Watanabe M, 2022 [22]	To investigate the impact of rapid weight loss on the adaptive immune response in subjects with morbid obesity	Italy	cross-sectional study	21 participants BMI ≥ 35 kg/m^2^ with at least one obesity-related complication or BMI ≥ 40 kg/m^2^ alone; Age: 51.50(41.50, 55.25)	5 to 7 weeks between the two vaccine doses	BNT162b2	Vaccine Efficacy: A high baseline BMI was correlated with a poor immune response	Obesity was associated with a reduced adaptive response to an mRNA COVID-19 vaccine
7	Tubjaroen, 2022 [14]	To evaluate immunogenicity among children with liver transplants and obesity following two doses of the BNT162b2 vaccine	Thailand	cohort study	*n* = 68 (Obese group: *n* = 24; LTRs group: *n* = 12; control group: *n* = 32); Age:14.9 ± 1.7	27.1 ± 3.2 days after the second dose	BNT162b2	Vaccine Efficacy: SARS-CoV-2 antibody levels at approximately four weeks following the second dose of the BNT162b2 vaccine in obese adolescents were lower than those in the control participants with normal weight	Obese adolescents showed low antibody response to the BNT162b2 vaccine
8	Yamamoto S, 2022 [23]	To investigate the impact of obesity on antibody response to a COVID-19 vaccine	Japan	cohort study	healthcare workers (*n* = 2435); Age:36.6(27.6,47.6)	2 months after the in-house vaccination program	BNT162b2	Vaccine Efficacy: Spike IgG antibody titers tended to decrease with increasing BMI in men	Higher BMI was associated with lower titers of SARS-CoV-2 spike antibodies in men, but not in women
9	Kara Z, 2022 [24]	To investigate the spike-protein receptor-binding domain antibody titers against BNT162b2mRNA and inactivated SARS-CoV-2 (CoronaVac) vaccines in people with severe obesity	UK	cohort study	study group (BMI ≥ 40 kg/m^2^, *n* = 124) normal weight control group (BMI 18.5–24.9 kg/m^2^, *n* = 166)	4th week and after 2nd dose of vaccination	(1) BNT162b2 (2) Corona Vac vaccines	Vaccine Efficacy: In 220 subjects (no prior infection) vaccinated with BNT162b2 or CoronaVac, the antibody titers against the SARS-CoV-2 spike antigen of patients with severe obesity were significantly lower than those of normal weight controls	Patients with severe obesity generated significantly reduced antibody titers against SARS-CoV-2 spike antigen after CoronaVac and BNT162b2 vaccines compared to people with normal weight
10	Pellini R, 2021 [25]	To analyze the antibody titer response 7 days after the second dose of a vaccine	Italy	cohort study	248 healthcare workers; Age: 47 (range 23–69)	baseline and 7 days after BNT162b2 booster dose	BNT162b2	Vaccine Efficacy: BMI had no statistically significant association with the geometric mean concentration of antibodies	BMI did not seem to be associated with a difference in immune response to the vaccine
11	Piernas C, 2022 [26]	To examine the association between BMI and COVID-19 vaccine uptake, vaccine effectiveness, and risk of severe COVID-19 outcomes after vaccination	UK	cohort study	9,171,524 participants Age: 52 ± 19	days from 8 December 2020	(1) ChAdOx-nCov19 (2) BNT162b2 (3) mRNA1273	Vaccine Efficacy: For COVID-19 test positivity, there was a linear association with BMI after the first dose, an exponential association after the second dose, and an inverse U-shaped association after the third dose, with significantly lower HRs at very low and very high BMI levels. Vaccine Safety: In the vaccinated cohort, there were significant linear associations of BMI with COVID-19 hospitalization and death after the first dose, and there were J-shaped associations after the second dose	In the vaccinated cohort, there were increased risks of severe COVID-19 outcomes for people with underweight or obesity compared with the vaccinated population with a healthy weight
12	Watanabe M, 2022 [13]	To explore variables associated with the serological response following an mRNA COVID-19 vaccine	Italy	cohort study	86 healthcare workers; Central obesity (*n* = 53); Age:29 ± 17	(1) Before the first inoculation (2) 1 and 4 weeks after the second inoculation	BNT162b2	Vaccine Efficacy: Higher waist circumference was associated with lower antibody titers Vaccine Safety: Higher waist circumference, waist-to-hip ratio, BMI, and body fat were not associated with more adverse events	Central obesity was associated with lower antibody titers following COVID-19 vaccination
13	Iguacel I, 2021 [27]	To study the association between weight status and reported side-effects	Spain	cross-sectional study	2136 adults	from 6 May to 9 June 2021	Pfizer, Moderna, and AstraZeneca/Vaxzevria	Vaccine Safety: Most side-effects were reported at a higher percentage in those who were underweight or normal weight compared to overweight or obese	A nonoverweight status was associated with a higher risk of presenting fever ≥38°, vomiting, diarrhea, and chills compared to those who were overweight
14	Yoshida, 2022 [28]	To investigate the type and frequency of adverse reactions in healthy and allergic disease individuals aged 5–11 years over the first seven days following the first and second BNT162b2 vaccinations	Japan	cohort study	*n* = 421; Mean age: 8.8 ± 1.9;	Seven days after vaccination	BNT162b2	Vaccine Safety: Compared to ‘normal’ individuals, ‘thin’ and ‘overweight’ individuals did not have higher odds of experiencing adverse systemic reactions	BMI was not associated with adverse systemic reactions
15	Sutardi, 2022 [29]	To evaluate a comparison between the Sinovac and Pfizer vaccines for children and teenagers under 18 y in Indonesia and other factors that influenced it	Indonesia	Cross-sectional study	*n* = 400 (Sinovac 200, Pfizer 200); Age: 12–17 years old	1–6 months after vaccination	Sinovac vaccine and Pfizer vaccine	Vaccine Safety: BMI was related to side-effects after: 1st vaccination: fever, pain in the injection area, lost smell and taste; 2nd vaccination: sleepiness, fever; 1–3 months post-vaccination: menstrual problems; 4–6 months post-vaccination: menstrual problems (*p* < 0.05)	Vaccine recipients with a BMI of less than 25 had a higher risk of having side-effects from the COVID-19 vaccine

Abbreviations: AO, abdominal obesity; BMI, body mass index; CI, confidence interval; LTRs, liver transplant recipients.

## Data Availability

The datasets generated during and/or analyzed during the current study are available from the corresponding author on reasonable request.

## Supplementary Materials

The following supporting information can be downloaded at https://www.mdpi.com/article/10.3390/vaccines11050996/s1, File S1: Search Strategies; Table S1: Risk-of-Bias Assessment using the Joanna Briggs Institute (JBI) Critical Appraisal Checklist for Cohort Studies; Table S2: Risk-of-Bias Assessment using the Joanna Briggs Institute (JBI) Critical Appraisal Checklist for Cross-Sectional Studies.
