# Supplementary material for: Association between Overweight/Obesity and the Safety and Efficacy of COVID-19 Vaccination: A Systematic Review
Source: Vaccines (Basel). 2023 May 17;11(5):996. doi: 10.3390/vaccines11050996 (PMC10223724; doi:10.3390/vaccines11050996)
Supplement: Supplementary file 1 [file vaccines-11-00996-s001.zip › vaccines-2312045-supplementary.pdf]

File S1: Search Strategies

Medline

(exp COVID-19 Vaccines/OR ((COVID-19/OR SARS-CoV-2/) AND (Vaccination/OR exp Vaccines/)) OR (((SARS-CoV-2 OR covid* OR coronavir*) ADJ3 vaccin*)).ab, ti. OR (((SARS-CoV-2 OR covid* OR coronavir*) AND vaccin*)).ti.) AND (exp Obesity/OR Body Mass Index/OR Body Weight/OR Anthropometry/OR body fat percentage/OR Waist Circumference/OR Waist-Hip Ratio/OR Waist-Height Ratio/OR Body Composition/OR Body Fat Distribution/OR ((Risk Factors/OR Risk/) AND ("Drug-Related Side Effects and Adverse Reactions"/OR Safety/OR Vaccine Efficacy/)) OR (obesit* OR obese* OR overweight OR bmi OR body-mass* OR body-weight* OR anthropometr* OR (waist ADJ3 (hip OR circumfere* OR ratio*)) OR (hip ADJ3 circumfere*) OR (weight ADJ3 height) OR body-fat OR fat-mass OR adipos* OR ((risk* OR factor*) ADJ6 (safet* OR efficac* OR effectiv*))).ab, ti.)

Embase.com

('SARS-CoV-2 vaccine'/exp OR (('coronavirus disease 2019'/exp OR 'Severe acute respiratory syndrome coronavirus 2'/exp) AND (vaccination/de OR vaccine/exp)) OR (((SARS-CoV-2 OR covid* OR coronavir*) NEAR/3 vaccin*)):ab, ti OR (((SARS-CoV-2 OR covid* OR coronavir*) AND vaccin*)):ti) AND (obesity/de OR 'body mass'/de OR 'body weight disorder'/de OR 'body weight'/de OR anthropometry/de OR 'anthropometric parameters'/de OR 'body fat percentage'/de OR 'waist circumference'/de OR 'waist hip ratio'/de OR 'waist to height ratio'/de OR 'weight height ratio'/de OR 'total body fat'/de OR 'hip circumference'/de OR 'fat mass'/de OR 'body composition'/de OR 'body adiposity index'/de OR 'body fat distribution'/de OR 'body fat percentage'/de OR (('risk factor'/de OR risk/de) AND ('adverse event'/de OR safety/de OR 'efficacy parameters'/de)) OR (obesit* OR obese* OR overweight OR bmi OR body-mass* OR body-weight* OR anthropometr* OR (waist NEAR/3 (hip OR circumfere* OR ratio*)) OR (hip NEAR/3 circumfere*) OR (weight NEAR/3 height) OR body-fat OR fat-mass OR adipos* OR ((risk* OR factor*) NEAR/6 (safet* OR efficac* OR effectiv*))):ab, ti)

Web of science

TS=(((((SARS-CoV-2 OR covid* OR coronavir*) NEAR/2 vaccin*)) OR (((SARS-CoV-2 OR covid* OR coronavir*) AND vaccin*)):ti) AND ((obesit* OR obese* OR overweight OR bmi OR body-mass* OR body-weight* OR anthropometr* OR (waist NEAR/2 (hip OR circumfere* OR ratio*)) OR (hip NEAR/2 circumfere*) OR (weight NEAR/2 height) OR body-fat OR fat-mass OR adipos* OR ((risk* OR factor*) NEAR/5 (safet* OR efficac* OR effectiv*))))))

Cochrane

((((SARS NEXT CoV NEXT 2 OR covid* OR coronavir*) NEAR/3 vaccin*)):ab, ti OR (((SARS NEXT CoV NEXT 2 OR covid* OR coronavir*) AND vaccin*)):ti) AND ((obesit* OR obese* OR overweight OR bmi OR body NEXT mass* OR body NEXT weight* OR anthropometr* OR (waist NEAR/3 (hip OR circumfere* OR ratio*)) OR (hip NEAR/3 circumfere*) OR (weight NEAR/3 height) OR body NEXT fat OR fat NEXT mass OR adipos* OR ((risk* OR factor*) NEAR/6 (safet* OR efficac* OR effectiv*))):ab, ti)

Google scholar

"SARS-CoV-2"|covid|coronavirus vaccination|vaccine obesity|obese|overweight|bmi|"body-mass weight"|"waist hip|circumference"|"hip circumference"|"body-fat"|"fat-mass"

Table S1. Risk-of-Bias Assessment using the Joanna Briggs Institute (JBI) Critical Appraisal Checklist for Cohort Studies.

Study	1. Two Groups Similar and Recruited from the Same Population?	2. Were the Exposures Measured Similarly, to Assign People to Both Exposed and Unexposed Groups?	3. Was the Exposure Measured in a Valid and Reliable Way?	4. Were Confounding Factors Identified?	5. Were Strategies to Deal with Confounding Factors Stated?	6. Were the Groups/ Participants Free of the Outcome at the Start of the Study (or at the Moment of Exposure)?	7. Were the Outcomes Measured in a Valid and Reliable Way?	8. Was the Follow-Up Time Reported and Sufficient to Be Long Enough for Outcomes to Occur?	9. Was Follow-Up Complete, and If Not, Were the Reasons to Loss of Follow-Up Described and Explored?	10. Were Strategies to Address Incomplete Follow-Up Utilized?	11. Was Appropriate Statistical Analysis Used?	% Yes	Risk of bias
Malavazos AE,2022	Yes	Yes	Yes	Unclear	No	Yes	Yes	Yes	Yes	No	Yes	73%	Low
Gaborit B,2023	Yes	Yes	Yes	Yes	No	Yes	Yes	Yes	Yes	Unclear	Yes	82%	Low
Zhu Q,2022	Yes	Yes	Yes	Unclear	No	Yes	Yes	Yes	Yes	No	Yes	73%	Low
Tubjaroen, 2022	Yes	Yes	Yes	Unclear	No	Yes	Yes	Yes	Yes	No	No	64%	Moderate
Yamamoto S, 2022	Yes	Yes	Yes	Yes	No	Unclear	Yes	Yes	Yes	Yes	Yes	82%	Low
Kara Z, 2022	Yes	Yes	Yes	Unclear	No	Yes	Yes	Yes	No	No	Yes	64%	Moderate
Pellini R, 2021	Yes	Yes	Yes	Unclear	No	Yes	Yes	Yes	Yes	Unclear	Yes	73%	Low
Piernas C, 2022	Yes	Yes	Yes	Yes	Yes	Unclear	Yes	Unclear	Yes	Yes	Yes	82%	Low
Watanabe M, 2022	Yes	Yes	Yes	Unclear	No	Yes	Yes	Yes	No	No	Yes	64%	Moderate
Yoshida, 2022	No	Unclear	Yes	Yes	Yes	Yes	Yes	Yes	No	No	Yes	73%	Low

Notes: The risk of bias was ranked as high when the study reached 49% "yes" scores, moderate when the study reached 50 to 69% "yes" scores, and low when the study reached more than 70% "yes" scores.

Table S2. Risk-of-Bias Assessment using the Joanna Briggs Institute (JBI) Critical Appraisal Checklist for Cross-Sectional Studies.

No.	Checklist questions	Faizo AA, 2023	Lee SW, 2021	Iguacel I, 2021	Sutardi, 2022	Watanabe M, 2022
1.	Were the criteria for inclusion in the sample clearly defined?	Yes	Yes	Yes	Yes	Yes
2.	Were the study subjects and the setting described in detail?	Unclear	Yes	No	Yes	Yes
3.	Was the exposure measured in a valid and reliable way?	Yes	Yes	Yes	Yes	Yes
4.	Were objective, standard criteria used for measurement of the condition?	Yes	Yes	Yes	Unclear	Yes
5.	Were confounding factors identified?	Unclear	Unclear	Yes	Unclear	No
6.	Were strategies to deal with confounding factors stated?	No	No	Unclear	No	No
7.	Were the outcomes measured in a valid and reliable way?	Yes	Yes	Yes	Unclear	Yes
8.	Was appropriate statistical analysis used?	Yes	Yes	Yes	No	Yes
	% Yes	63%	75%	75%	38%	75%
	Risk of bias	Moderate	Low	Low	High	Low

Notes: The risk of bias was ranked as high when the study reached 49% "yes" scores, moderate when the study reached 50 to 69% "yes" scores, and low when the study reached more than 70% "yes" scores.
